# Delta/Notch-like Epidermal Growth Factor-Related Receptor (DNER), a Potential Prognostic Marker of Gastric Cancer Regulates Cell Survival and Cell Cycle Progression

**DOI:** 10.3390/ijms241210077

**Published:** 2023-06-13

**Authors:** Han Thi Ngoc To, Ji-Hong Park, Jeong Won Kim, Dongchul Kang

**Affiliations:** 1 Ilsong Institute of Life Science, Hallym University, Beodeunaru-ro 55, Yeongdeungpo-gu, Seoul 07247, Republic of Korea; ngochantt.90@gmail.com (H.T.N.T.); flowjh@hallym.ac.kr (J.-H.P.); 2 Department of Biomedical Gerontology, Hallym University Graduate School, Chuncheon 24252, Republic of Korea; 3 Department of Pathology, Kangnam Sacred Heart Hospital, College of Medicine, Hallym University, Seoul 07441, Republic of Korea; jwkim@hallym.or.kr

**Keywords:** gastric cancer, spheroid formation, DNER, Notch signaling, NICD, TGF-β, apoptosis, cell cycle, p21^cip/waf^, p53

## Abstract

Upregulation of the expression of Delta/notch-like epidermal growth factor-related receptor (DNER) and its oncogenic role have been reported in several cancers, including gastric, breast, and prostate cancers. This study aimed to investigate the oncogenic role of DNER and the mechanisms behind its oncogenic role in gastric cancer. Analysis of the RNASeq data of gastric cancer tissues obtained from the TCGA database revealed that the expression of DNER was associated with the pathology of advanced gastric cancer and the prognosis of patients. DNER expression was increased upon stem cell-enriching cancer spheroid culture. Knockdown of DNER expression inhibited cell proliferation and invasion, induced apoptosis, enhanced chemosensitivity, and decreased spheroid formation of SNU-638 gastric cancer cells. DNER silencing elevated the expression of p53, p21^cip/waf^, and p27, and increased G_1_ phase cells at the expense of S phase cells. Knockdown of p21^cip/waf^ expression in the DNER-silenced cells partially restored cell viability and S phase progression. DNER silencing also induced the apoptosis of SNU-638 cells. While both cleaved caspases-8 and 9 were detected in adherent cells, only cleaved caspase-8 was found to have increased in spheroid-cultured cells, suggesting a distinct activation pattern of caspase activation depending on the growth condition. Knockdown of p53 expression rescued the DNER-silenced cells from apoptosis and partially restored cell viability. In contrast, overexpression of the Notch intracellular domain (NICD) decreased the expression of p53, p21^cip/waf^, and cleaved caspase-3 in DNER-silenced cells. Moreover, NICD expression fully reverted the cell viability reduction, arrest in the G_1_ phase, and elevated apoptosis caused by DNER silencing, thereby suggesting activation of Notch signaling by DNER. Expression of a membrane-unbound mutant of mDNER also decreased cell viability and induced apoptosis. On the other hand, TGF-β signals were found to be involved in DNER expression in both adherent and spheroid-cultured cells. DNER could therefore be a link connecting TGF-β signaling to Notch signaling. Taken together, DNER regulates cell proliferation, survival, and invasive capacity of the gastric cancer cells through the activation of Notch signaling, which may facilitate tumor progression into an advanced stage. This study provides evidences suggesting that DNER could be a potential prognostic marker, a therapeutic target, and a drug candidate in the form of a cell-free mutant.

## 1. Introduction

Gastric cancer is the fifth most commonly reported cancer, and the fourth leading cause of cancer-related mortality worldwide [1]. These facts underscore the significant impact of gastric cancer on both public health and the well-being of patients, accompanied with the urgent need for effective treatments. Given the complex nature of gastric cancer, it is therefore imperative to gain a comprehensive understanding of the molecular mechanisms implicated in its development and progression in order to develop effective therapies.

Notch signaling plays a role in metazoan development and in the maintenance of adult tissue homeostasis [2]. Notch modulates the fate of neighboring cells through direct interactions of the membrane-bound Notch–Notch ligand. The precursor of Notch generates a heterodimeric receptor through cleavage at site 1 (S1) by furin-like convertases in the Golgi network [3,4]. This heterodimeric receptor translocates to the membrane at which it interacts with a Notch ligand on the neighboring cells through epidermal growth factor (EGF) repeats. The binding of the ligand-receptor stimulates the endocytosis of the ligand into the signaling cell, which results in the exposure of an extracellular proteolytic site, S2 [3,4]. Successive digestion by ADAM/TACE at S2 and by the γ-secretase complex at the intramembrane S3 site of the Notch releases a Notch intracellular domain (NICD) that translocates into the nucleus. NICD forms a complex with CSL (CBF1/Suppressor of Hairless/LAG-1) and MAML (Mastermind-like), leading to the transactivation of their target genes, including HES (hairy and enhancer of split) and HEY (HES related with YRPW motif). This relatively simple Notch signaling scheme can be further complicated by canonical CSL-dependent and non-canonical CSL-independent pathways. There are also DSL (delta-serrate-Lag-2)-containing canonical Notch ligands and DSL-lacking non-canonical Notch ligands [5].

In recent decades, Notch signaling pathways have emerged as a promising therapeutic target of cancer [6]. Activating mutations of Notch1 and aberrant activations of Notch were reported in T cell acute lymphoblastic leukemia and other solid cancers, including breast, colorectal, and pancreatic cancer, although loss-of-function mutations of Notch have been found in liver cancer and small cell lung cancer [7,8,9]. The Notch signaling pathway involves in tumor development and progression by modulating the expression of various Notch target genes, such as cyclin D1, NF-kB, and c-Myc [10,11,12]. Notch signaling was also reported to play a critical role in the maintenance of tumor stem cells in breast and pancreatic cancers [13,14]. Expression of Notch and Notch ligands were shown to be associated with the pathology and prognosis of gastric cancer patients [15,16,17]. Activation and overexpression of Notch1 promote gastric cancer progression through COX2 expression and the PTEN-ERK1/2 signaling axis, respectively [18,19]. Both canonical and non-canonical Notch signaling are involved in tumor development and progression [6,7].

Delta/Notch-like EGF receptor (DNER) is a DSL-lacking non-canonical Notch ligand. Although controversial, DNER was found to bind Notch1 at cell-cell contact and activate the Notch signaling pathway [20,21]. DNER has been found to play a crucial role in the development of the nervous system [22]. Specifically, the loss of DNER has been associated with abnormal glial morphology in cerebellar development [20]. DNER modulates polarity, synaptogenesis, and neuritogenesis during the development of the nervous system [23]. The association of DNER with cerebellar ataxia has been suggested following the identification of anti-DNER antibodies in lesions [24]. Furthermore, DNER has also been implicated as an oncogene, as it promotes the proliferation, migration, and invasion of various cancer cells. DNER has also been associated with cancer stemness and the progression of both breast cancer and prostate cancer [25,26]. Additionally, DNER has been linked to the proliferation and development of hepatocellular carcinoma [27]. Notably, DNER expression is significantly higher in cancer tissues compared to normal tissues, particularly in prostate cancer and breast cancer, and is associated with a poor prognosis [25,26,28].

DNER has been identified as a molecular marker that is associated with tumorigenesis and increased mortality in gastric adenocarcinoma patients [29]. In addition, analysis of the public database and 30 tumor tissues found that DNER expression was upregulated in gastric cancer tissues, and was associated with tumor size, lymph node metastasis, and a poor prognosis [28]. They also found that the knockdown of DNER expression inhibited proliferation, migration, and invasion, and induced the apoptosis of gastric cancer cells. Clearly, DNER appears to play a role in maintaining the proliferation and survival of cancer cells, which might therefore result in a poor prognosis for the cancer patient.

In this study, we examined the oncogenic characteristics of DNER in the progression of gastric cancer in more detail. The role of DNER in the maintenance of gastric cancer stem cells, and potential mechanisms involved in the regulation of proliferation and survival of gastric cancer cells was investigated by tumor spheroid cultures and analysis of the expression of the associated genes. The expression of DNER in tumor spheroid cultures and its signaling pathway were also analyzed and were found to be upregulated through the TGF-β pathway. In addition, the growth inhibitory effect of secreted DNER was examined to assess its potential for tumor suppression.

## 2. Results

### 2.1. Pathological Association of DNER Expression in Gastric Cancer

**Figure 1:**
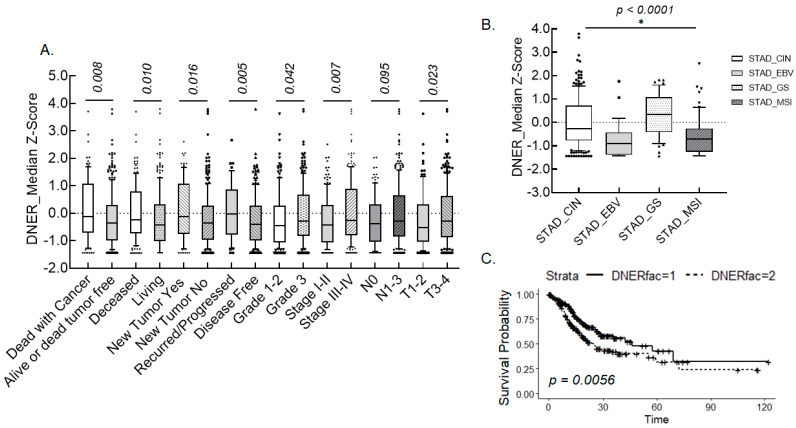
**Association of DNER expression with various clinicopathological parameters and survival probability in gastric cancer**. (**A**). Distribution of the median z-score of DNER expression depending on each category of the indicated clinicopathological parameters. The probabilities between the categories were calculated by the Wilcoxon rank sum test. (**B**). The Kaplan–Meier cumulative survival rate curve according to the expression of DNER in gastric cancer tissue. The solid line includes patients with DNER expression in the lower half (fac = 1), and the dashed line includes patients with DNER expression in the upper half (fac = 2). (**C**). Distribution of the median z-score of DNER expression depending on the molecular subtypes of stomach adenocarcinoma (STAD). CIN, chromosomal instability; EBV, Epstein–Barr virus; GS, genomically stable; and MSI, microsatellite instability. The probabilities were calculated using the Kruskal–Wallis test. * represents *p* < 0.05.

A clinicopathological association of DNER expression in gastric cancer tissue was analyzed with RNASeq data downloaded from the TCGA database. DNER expression was significantly associated with tumor stage (*p* = 0.007), T status (*p* = 0.023), tumor grade (*p* = 0.042), disease-free status (*p* = 0.005), new tumor event after initial treatment (*p* = 0.016), overall survival (*p* = 0.010) and disease-specific survival (*p* = 0.008) (Figure 1A). DNER expression was moderately associated with N status (*p* = 0.095) between the N0 stage and N1/2/3 stage but was found to be significantly associated with between the N0/1 stage and the N2/3 stage (*p* = 0.020). Median DNER expression was found to be higher in patients with recurred/progressed disease, new tumors after initial treatment, tumor-specific death, and non-specific death. Moreover, median DNER expression was also found to be greater in tumor tissues of grade 3, tumor stage III/IV, and T status III/IV, compared to grade 1/2, tumor stage I/II, and T status I/II, respectively. In addition, DNER was found to have expressed differently among the molecular subtypes of gastric cancer defined by TCGA analysis (Figure 1B). DNER expression was the highest in the genomically stable subtype (GS), while the Epstein–Barr virus-positive (EBV) and microsatellite instable (MSI) subtypes showed similarly low levels of DNER expression. The cumulative survival probability of patients with higher DNER expression levels was found to be significantly lower in Kaplan–Meier analysis (*p* = 0.0056, Figure 1C). Overall, these findings indicate that DNER expression should be significantly associated with both gastric cancer progression and worse prognosis of patients.

### 2.2. DNER Expression Increased in the Tumor Spheroid Culture of Gastric Cancer Cells

**Figure 2:**
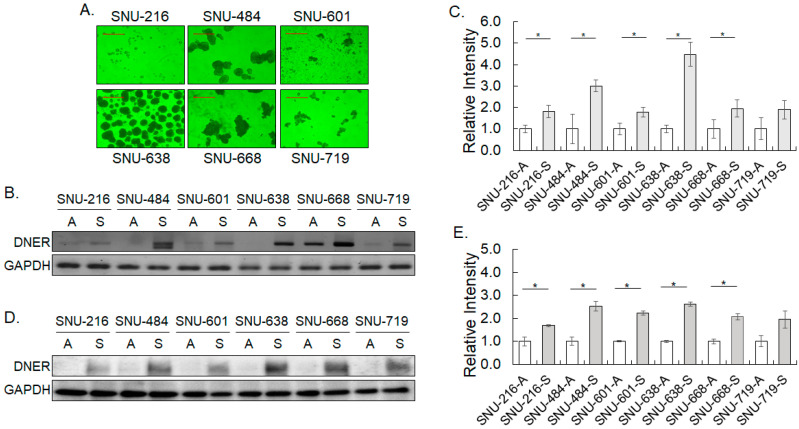
**DNER expression increases in the spheroid of the gastric cancer cells.** (**A**) Phase contrast microscope images (20×) of spheroid formation of the gastric cancer cell lines, SNU-216, SNU-484, SNU-601, SNU-638, SNU-668, and SNU-719, after 14 days of spheroid culture. A scale bar of 500 µm. (**B**) The transcript level of DNER in the 14-day spheroid-cultured cells was determined by RT-PCR. GAPDH transcript level was measured to monitor the input RNA amount. (**C**) The transcript level of DNER normalized against that of GAPDH. The intensity of each cDNA band was quantitated with ImageJ software. Data shown are folds over the level in the adherent cells. (**D**) The protein level of DNER in the 14-day spheroid-cultured cells was determined with Western blotting. GAPDH protein level was measured to monitor protein loading. (**E**) The protein level of DNER normalized against that of GAPDH. The intensity of each protein band was quantitated with ImageJ software. Data shown are folds over the level in the adherent cells. All quantitative data shown here are the mean ± SD of three independent experiments. * represents *p* < 0.05.

The expression of DNER was compared between cells grown in adherent condition and in non-adherent tumor spheroid culture condition that has been widely employed to enrich tumor stem cells or stem cell-like cells. Spheroid formation varied among the six gastric cancer cell lines (SNU-216, SNU-484, SNU-601, SNU-638, SNU-668, and SNU-719, respectively) (Figure 2A). SNU-484, SNU-638, and SNU-668 formed large clusters of cells with distinct spheroid morphology, while SNU-601 and SNU-719 formed cell clusters of smaller sizes. SNU-216 cells did not grow well under this condition. DNER expression increased in all six cell lines grown in the spheroid culture condition at both mRNA and protein levels (Figure 2B–E). RT-PCR results showed that the DNER transcript level significantly increased by approximately 2~4-fold in the spheroid culture of SNU-216, SNU-484, SNU-601, SNU-638, and SNU-668 (*p* = 0.01, *p* = 0.008, *p* = 0.01, *p* = 0.0005 and *p* = 0.04, respectively, Figure 2B,C). DNER protein levels also increased by 1.6-fold in SNU-216 spheroids (*p* = 0.04) and by more than 2.5-fold in SNU-484, SNU601, SNU-638, and SNU-668 spheroid cells (*p* = 0.010, *p* = 0.001, *p* = 0.05, and *p* = 0.049, respectively, Figure 2D,E). Although statistical significance was compromised, mRNA and protein levels of DNER also appeared to increase in SNU-719. These results demonstrate that DNER expression at both the mRNA and protein levels increase in the cells grown under non-adherent spheroid cultured conditions, regardless of spheroid formation.

### 2.3. DNER and NICD Expression Increased in Spheroid Formation

**Figure 3:**
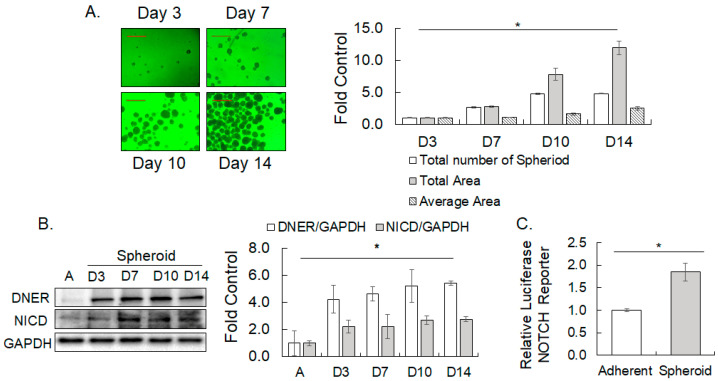
**The expression of DNER and NICD, and Notch reporter activity in spheroid-cultured SNU-638 cells.** (**A**) Phase contrast microscope images (20×) of spheroid formation at indicated time points. The spheroid analysis was performed using ImageJ software. Data shown are folds over the day 3 results. A scale bar of 500 µm. (**B**) The expression of DNER and NICD at the indicated time points after the spheroid culture was examined using Western blotting. GAPDH was used as a loading control. The band intensity was quantitated with ImageJ software. Data shown are folds over the level in the adherent cells (**A**). (**C**) Notch reporter activity was compared between adherent and spheroid-cultured cells for three days by the dual luciferase assay. Data shown are folds over the level in the adherent cells. All quantitative data shown here are the mean ± SD of three independent experiments. * represents *p* < 0.05.

Since SNU-638 formed better demarcated spheroids compared to the other gastric cancer cells, the expression and function of DNER in spheroid formation were analyzed further with the SNU-638 cells. Spheroid formation of SNU-638 increased over time in the culture, with significant increases observed in the total number and total area of spheroids after 7 and 14 days compared to day 3 (*p* = 3.31 × 10^−7^ and *p* = 0.00003, respectively) (Figure 3A). The average area of the spheroids also significantly increased after 10 and 14 days of culture (*p* = 0.002 and *p* = 0.0006, respectively) (Figure 3A), indicating the progressive development of the spheroids. DNER expression was elevated by 4.2-fold (*p* = 0.0005) after the first 3 days of spheroid culture compared to the adherent cells and maintained this increased level until day 14 (Figure 3B). An increase in DNER expression at an early time point of spheroid culture suggests its role in the growth and survival of cells at an early time of spheroid culture or in the nucleation of the spheroid. To examine whether the increase in DNER expression was accompanied with the activation of the Notch signaling pathway, the level of NICD was determined by Western blotting. NICD level was found to have increased by 2.1-fold after 3 days of spheroid culture compared to the adherent cells (*p* = 0.008, Figure 3B), and remained elevated until day 14. Furthermore, Notch-dependent reporter activity was upregulated by 1.8-fold (*p* = 0.001) in 3-day spheroid culture compared to the adherent cells (Figure 3C). Thus, the upregulation of DNER expression was accompanied with an increase in NICD expression and Notch-dependent reporter activity. These results suggest that DNER might function as a Notch ligand [20], and that the Notch signaling pathway activated by DNER might be involved in the formation of spheroids in the culture condition.

### 2.4. Silencing DNER Expression Reduced Spheroid Formation and Cell Viability

**Figure 4:**
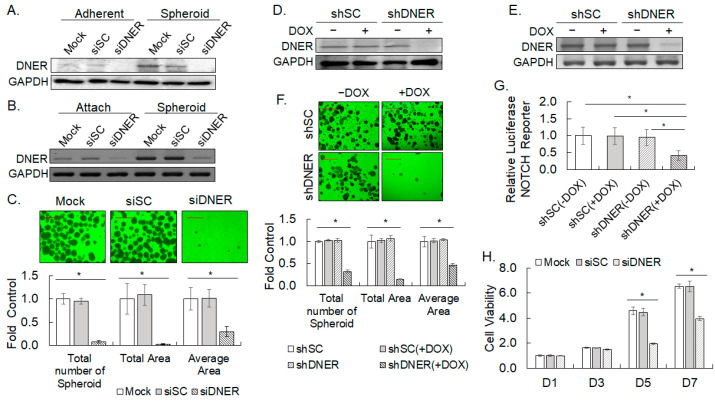
**Knockdown of DNER expression inhibits spheroid formation, Notch reporter activity, and cell viability.** (**A**,**B**) The DNER protein level (**A**) and transcript level (**B**) in cells transfected with siSC (scrambled siRNA) or siDNER were measured by Western blotting and RT-PCR, respectively. Mock cells were treated with the transfection reagent only and were cultured for the same period. GAPDH was used as the loading control in Western blotting and as the RNA amount control for RT-PCR. (**C**) Phase contrast microscope images (20×) of spheroid-cultured SNU-638 cells transfected with siSC and siDNER for 14 days. The area or the total number of spheroids was analyzed using ImageJ software. Data shown are folds over the mock control. A scale bar of 500 µm. (**D**,**E**) The DNER protein level (**D**) and transcript level (**E**) in SNU-638 cells transduced with lentivirus expressing shSC (scrambled shRNA) or shDNER in a DOX-inducible pattern. The lentivirus-transduced cells were selected with puromycin (2 µg/mL) treatment for seven days and were then treated with DOX (10 ng/mL) for three days. GAPDH was used as the loading control in Western blotting and as the RNA amount control for the RT-PCR. (**F**) Phase contrast microscope images (20×) of spheroid-cultured SNU-638 cells expressing shSC and shDNER for 14 days. Quantitative data were obtained and analyzed as described above (**C**). (**G**) The Notch reporter activities of shSC and shDNER cells were measured by the dual luciferase assay. DOX was treated to cells transfected with the Notch reporter for three days. Data shown are the folded increases over the untreated shSC cells. (**H**). Cell viability of siSC- or siDNER-transfected cells was measured by the MTT assay on 1, 3, 5, and 7 days post-transfection. Data shown are fold increases over the viability of day 1 mock-transfected cells. All quantitative data shown here are the mean ± SD of three independent experiments. * represents *p* < 0.05.

In order to investigate the effect of DNER on the spheroid formation of gastric cancer cells, the expression of DNER was silenced by the transfection of siDNER and the transduction of DOX-inducible shDNER lentivirus into SNU-638 cells. Knockdown of DNER expression by siDNER transfection and lentiviral shDNER expression was confirmed with Western blotting (Figure 4A,D, respectively) and RT-PCR (Figure 4B,E, respectively). Silencing DNER expression by siDNER and shDNER led to a significant reduction in spheroid formation of the gastric cancer cells (Figure 4C,F, respectively). The total number of spheroids and the total spheroid area of the siDNER cells was found to have decreased by 90% compared to the scrambled siRNA (siSC)-transfected control (*p* = 0.001 and *p* = 0.007, respectively). The average spheroid area of the siDNER cells decreased by over 60% compared to the siSC controls (*p* = 0.01, Figure 4C). Similar results were observed in cells expressing DOX-induced shDNER (Figure 4F), with a 67.8% reduction in total spheroid number (*p* = 0.0010), an 80% reduction in total spheroid area (*p* = 0.00027), and a 54% reduction in average spheroid area (*p* = 0.0016), respectively. Meanwhile, Notch reporter activity was reduced by 59% (*p* = 0.03) in DOX-induced shDNER expressing cells compared to the DOX-treated shSC cells (Figure 4G). Reduction in spheroid formation was also observed in DNER-silenced SNU-484 and SNU-668 cells (Supplemental Figure S1). To further determine the role of DNER in spheroid formation, the cell viability of the DNER-silenced cells was measured by the MTT assay. Cell viability of the siDNER-transfected cells was reduced by 57% in 5 days (*p* = 0.00012) and by 40% in 7 days (*p* = 0.00072), respectively, compared to the siSC-transfected cells (Figure 4H). In summary, silencing DNER expression clearly reduced spheroid formation and cell viability, which suggests that DNER might be involved in spheroid formation through the regulation of cell viability. Activation of Notch signaling by DNER in this process was inferred by a decrease in Notch reporter activity by DNER silencing.

### 2.5. Silencing DNER Expression Inhibited Cell Cycle Progression at the G_1_/S Phase

**Figure 5:**
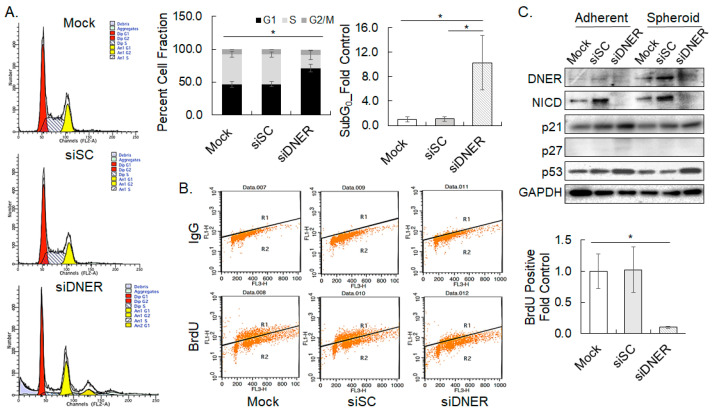
**Silencing DNER inhibits cell cycle progression of the gastric cancer cells.** (**A**) Flow cytometric analysis of cells transfected with siSC or siDNER and stained with PI. Cell fractions in each cell cycle phase were measured with ModFit LT^TM^ software. (**B**) Flow cytometric analysis of 2 h pulse BrdU-incorporated cells to detect DNA neosynthesis. Data shown are folds over the mock transfected cells. (**C**) The expression of indicated cell cycle-related proteins was examined using Western blotting. Band intensity was quantitated and analyzed as above (Figure 2D). All quantitative data shown here are the mean ± SD of three independent experiments. * represents *p* < 0.05.

The effect of DNER expression on cell cycle progression and apoptosis was analyzed by flow cytometry and Western blotting as DNER silencing was found to reduce cell viability. Flow cytometric analysis of the PI (propidium iodide)-stained cells revealed that DNER silencing significantly increased cells in the G_1_ phase by 1.55-fold (*p* = 0.0035) at the expense of a 57% reduction of S phase cells (*p* = 0.0056) compared to the siSC-transfected cells. The fraction of G_2_/M phase cells remained unchanged compared to the siSC control. Moreover, sub-G_0_ cells became evident, and took up to 11.4% (9.7-fold over siSC) of the cell population in siDNER cells (*p* = 0.023) (Figure 5A). In addition, pulsed BrdU incorporation representing newly synthesized DNA also decreased significantly by 90% in DNER-silenced cells (*p* = 0.004) (Figure 5B). G_1_ phase arrest of the cell cycle by silencing DNER expression has been implied here by the increase in G_1_ phase cells with a concomitant decrease in S phase cells, and the reduction of BrdU-positive cells. Consistent with these findings, the expression of the cell cycle regulators p21^cip/waf^, p27, and p53 was noticeably upregulated by 2.20-fold (*p* = 0.01), 5.3-fold (*p* = 0.027), and 3.1-fold (*p* = 0.042) in adherent siDNER cells, respectively, and by 2.06-fold (*p* = 0.001), 1.65-fold (*p* = 0.013), and 2.12-fold (*p* = 0.0097) in spheroid-cultured siDNER cells, respectively (Figure 5C). In addition, the level of NICD was significantly decreased by silencing DNER up to 28% in the adherent culture (*p* = 0.019) and 44% in the spheroid culture (*p* = 0.005) of the siSC control, respectively (Figure 5C). Overall, DNER silencing suppresses cell proliferation by preventing the cells from S phase entry, possibly through the downregulation of NICD and the upregulation of the CDK inhibitors p21^cip/waf^ and p27 that are transcriptional targets of p53 [30,31,32].

### 2.6. Silencing DNER Expression Increased Apoptotic Cell Death

**Figure 6:**
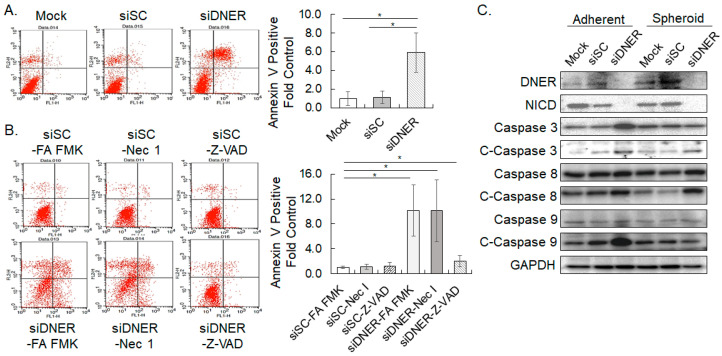
**Knockdown of DNER expression elicits apoptosis.** (**A**) Flow cytometric analysis to measure Annexin V binding to cells transfected with siSC or siDNER. Data shown are fold increases in the Annexin V-positive cells over the mock control. (**B**) Flow cytometric analysis to measure Annexin V binding to siDNER-transfected cells treated with inhibitors of necrosis (Necrostatin-1, Nec 1) and apoptosis (Z-VAD-FMK). Data shown are fold increases in the Annexin V-positive cells over the control treatment (siSC-FA-FMK). (**C**) The level of caspases-3, -8, -9, and their cleaved fragments (marked as C-) was examined by Western blotting. GAPDH was used for loading control in the western blotting. All quantitative data shown here are the mean ± SD of three independent experiments. * represents *p* < 0.05.

Flow cytometric analysis of Annexin V binding was applied to determine the cell death of the DNER-silenced gastric cancer cells. Annexin V binding cells were elevated by 5.4-fold in the siDNER cells compared to the siSC cells (*p* = 0.02), indicating that cell death was induced by DNER silencing (Figure 6A). Treatment with the pan-caspase inhibitor Z-VAD, but not necrostatin rescued DNER-silenced cells from cell death by 88% (*p* = 0.0026, Figure 6B). Thus, the death of DNER-silenced cells was attributed to caspase-dependent apoptosis, not to necrosis. To further elucidate the specific caspases involved in the apoptotic process, the expression of caspase-3, caspase-8, caspase-9, and their cleaved fragments was examined using Western blot analysis (Figure 6C). Knockdown of DNER expression increased the protein levels of both the intact and cleaved caspases-3, -8, and -9. Notably, the level of cleaved caspase-3 was increased in both the adherent cells (3.61-fold, *p* = 0.0017) and spheroid-cultured cells (2.98-fold, *p* = 0.0005). The level of cleaved caspase-8 was also elevated in cells grown in both adherent and spheroid-cultured cells (1.70-fold, *p* = 0.0075; and 2.25-fold, *p* = 0.0023, respectively), while the increase in cleaved caspase-9 was not obvious in the spheroid-cultured cells (2.06-fold, *p* = 0.0098 for adherent cells; and 1.24-fold, *p* = 0.1576 for spheroid-cultured cells, respectively). Distinct caspases appear to be activated in the apoptosis of cells grown under different culture conditions. Collectively, DNER silencing promotes the caspase-dependent apoptotic cell death of the gastric cancer cells, and the activation of specific caspases seems to be dependent on the culture conditions.

**Figure 7:**
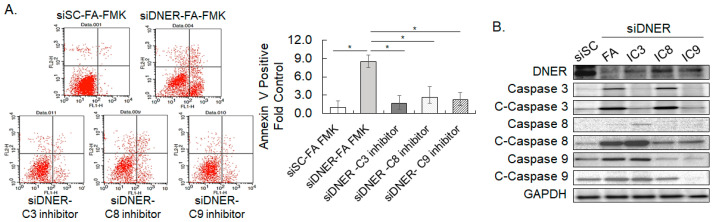
**Caspase activation pattern of DNER-silenced cells grown in adherent culture.** (**A**). Flow cytometric analysis to measure Annexin V binding to siDNER-transfected cells treated with the inhibitors of caspases-3 (Z-DEVD-FMK), -8 (Z-IETD-FMK), and -9 (Z-LEHD-FMK). Data shown are fold increases in the Annexin V-positive cells over the control treatment (siSC-FA-FMK). (**B**). The level of caspases-3, -8, -9, and their cleaved fragments (marked as C-) was examined by Western blotting. GAPDH was used as the loading control in the Western blotting. All quantitative data shown here are the mean ± SD of three independent experiments. * represents *p* < 0.05.

In order to further define the apoptotic pathway activated by DNER silencing, specific inhibitors of caspases-3, -8, and -9 were treated to the DNER-silenced cells grown under adherent conditions. Annexin V binding cells that were previously increased by DNER knockdown was reduced by all three caspase inhibitors (79.8% by Z-DEVD, 68.2% by Z-IETD, and 73.2% by Z-LEHD; *p* = 0.02, *p* = 0.008, and *p* = 0.03, respectively), denoting the involvement of all three caspases in this process (Figure 7A). Interestingly, activation patterns analyzed by caspase cleavage showed that the generation of cleaved caspase-3 was inhibited by the inhibitors of caspases-3 and -9 up to 33% (*p* = 0.0004) and 50% (*p* = 0.0006) compared to the FA-FMK control, respectively (Figure 7B). Caspase-8 inhibitor did not meaningfully suppress caspase-3 cleavage (85% of the control, *p* = 0.1745), although caspase-8 cleavage was clearly observed in DNER-silenced cells. Rather, the amount of cleaved caspase-8 was increased by caspase-3 inhibitor treatment. Considering that caspase-8 inhibitor also reduced Annexin V binding in flow cytometric analysis, these results were not reconciled with the well-established vertical caspase activation pathway between caspase-8 to caspase-3. However, these results clearly support the role of caspase-9 in the apoptosis of DNER-silenced cells grown in adherent culture.

### 2.7. Silencing DNER Expression Enhanced Chemosensitivity and Reduced Invasion of Gastric Cancer Cells

**Figure 8:**
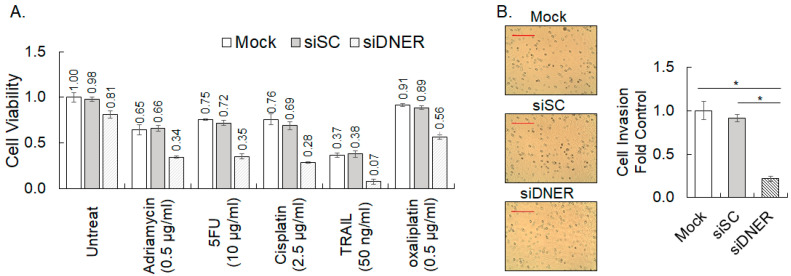
**Silencing DNER enhances the chemosensitivity and reduces the invasion of the gastric cancer cells.** (**A**) Cell viability of DNER-silenced cells treated with the indicated drugs for three days was measured using the MTT assay. Data shown are fold increases over the untreated mock control. (**B**) Transwell migration/invasion assay using Matrigel^®^-coated wells to measure the invasion of tumor cells. Four random microscopic fields were pictured (20×), and the image was analyzed with the ImageJ software to count the invaded cells. Data shown are fold increases over the mock-transfected cells. A scale bar of 500 µm. All quantitative data shown here are the mean ± SD of three independent experiments. * represents *p* < 0.05.

In order to investigate the role of DNER in the sensitivity of gastric cancer cells to various chemotherapeutic drugs and the tumor necrosis factor-related apoptosis-inducing ligand (TRAIL), cell viability of the siDNER-transfected cells treated with adriamycin, 5-fluorouracil (5-FU), cisplatin, TRAIL, and oxaliplatin was compared with the siSC-transfected cells. MTT assays of 3-day drug-treated cells showed that knockdown of DNER expression reduced viability significantly more than the additive effect of each individual treatment (Figure 8A). These findings highlight that silencing DNER enhances the chemosensitivity of the gastric cancer cells, indicating a collaborative interaction between the drug treatment and DNER silencing. The nature of this cooperativity should be determined further in detail. The role of DNER in gastric cancer progression was further analyzed with respect to invasion capacity. Invasion of the DNER-silenced cells was compared with the control cells by measuring the transwell migration of the cells through a Matrigel^®^-coated membrane. Knockdown of DNER significantly declined the number of migrated cells by 76% compared to the siSC control cells (*p* = 0.00002) (Figure 8B). These results coincide well with a previous report on the role of DNER in tumor cell invasion [28], thereby suggesting that DNER should contribute to gastric cancer progression by regulating their invasive capacity.

### 2.8. Overexpression of NICD Rescued DNER-Silenced Cells from Growth Arrest and Apoptosis

**Figure 9:**
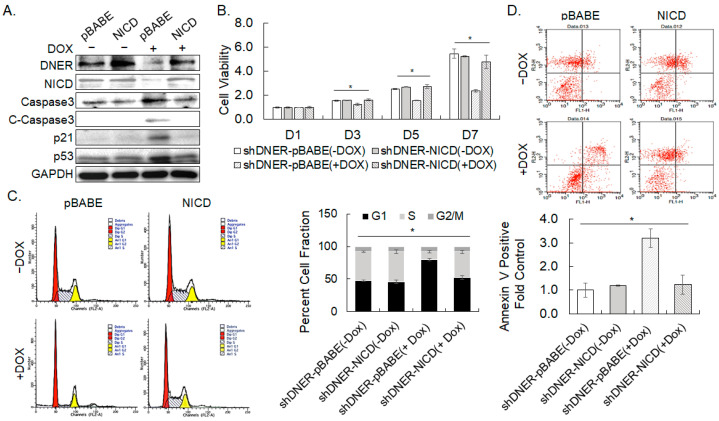
**Overexpression of NICD rescues shDNER cells.** (**A**) The level of the indicated proteins, including NICD was examined by Western blotting. The SNU-638 cells transduced with DOX-inducible shDNER were further transduced with the retrovirus of pBABE-zeo or pBABE-zeo-NICD and were treated with DOX for seven days. GAPDH was used as the loading control in the Western blotting. (**B**) The cell viability of the indicated cells was measured using the MTT assay after 1, 3, 5, and 7 days of DOX treatment. Data shown are fold increases over the DOX-untreated shDNER-pBABE cells. (**C**,**D**) Flow cytometric analysis of cell cycle progression (**C**) and Annexin V binding. (**D**) SNU-638 cells were transduced and treated as in Figure 9A. Cell fractions in each cell cycle phase was measured with ModFit LT^TM^ software. Data for Annexin V-positive cells are fold increases over DOX-untreated shDNER-pBABE cells. All quantitative data shown here are the mean ± SD of three independent experiments. * represents *p* < 0.05.

The downregulation of NICD at DNER silencing (Figure 5C and Figure 6C), and the reduction of Notch reporter activity in shDNER-expressing cells (Figure 4G) suggest that NICD generated by the activation of Notch signaling should be a functional mediator of DNER. In order to investigate the role of NICD in DNER-related cell cycle progression and survival, the effects of NICD overexpression was assessed in DNER-silenced gastric cancer cells by DOX-inducible shDNER expression. Overexpression of NICD in shDNER cells curtailed the protein expression of cleaved caspase-3 to 17% (*p* = 0.001), p21^cip/waf^ to 29% (*p* = 0.0003), and p53 to 67% (*p* = 0.036) of elevated level in the control shDNER cells (Figure 9A). The DNER protein level was also restored by the overexpression of NICD (Figure 9A). In addition, NICD overexpression fully recovered the cell viability of DOX-treated DNER-silenced cells to that of the control DOX-untreated shDNER cells in 5 and 7 days of culture (Figure 9B). The protein expression pattern and recovery of viability upon NICD overexpression suggest that NICD might reconstitute the growth potential and survival capacity reduced by DNER silencing. Therefore, the effect of NICD overexpression on cell cycle progression and apoptosis was analyzed with flow cytometry. NICD overexpression increased the S phase cell fraction up to 39.7% from 12.1% of DOX-treated shDNER cells (*p* = 0.00009), which was 86.5% of the level of DOX-untreated shDNER cells. Sub-G_0_ cells also decreased by 69% upon NICD overexpression compared to the shDNER cells without NICD expression (*p* = 0.05) (Figure 9C). Furthermore, Annexin V binding cells were also reduced by 62% in cells with overexpressed NICD compared to the shDNER cells without NICD expression (*p* = 0.003) and were comparable with DOX-untreated shDNER cells (Figure 9D). These findings indicate that the overexpression of NICD results in a nearly full recovery of cell cycle restriction and apoptosis caused by DNER silencing with shDNER expression. The fact that NICD overexpression reverses the effects of DNER knockdown on cell viability, apoptosis, and cell cycle progression strongly support that NICD generated from Notch signaling should be a major mediator of the DNER effect.

### 2.9. Depletion of p21^cip/waf^ Rescued DNER-Silenced Cells from Cell Cycle Arrest

**Figure 10:**
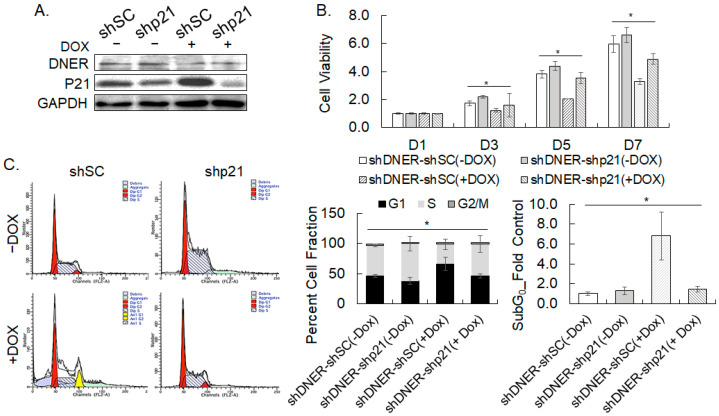
**Depletion of p21^cip/waf^ restores cell cycle progression in shDNER cells.** (**A**) The level of DNER and p21^cip/waf^ proteins was examined by Western blotting. The SNU-638 cells transduced with DOX-inducible shDNER were further transduced with lentivirus expressing shSC or shp21 and were treated with DOX for seven days. GAPDH was used as the loading control for the Western blotting. (**B**) The cell viability of the indicated cells was measured using the MTT assay after 1, 3, 5, and 7 days of DOX treatment. Data shown are fold increases over the DOX-untreated shDNER-shSC cells. (**C**) Flow cytometric analysis of cell cycle progression. SNU-638 cells were transduced and treated as in Figure 10A. Cell fractions in each cell cycle phase were measured with ModFit LT^TM^ software. All quantitative data shown here are the mean ± SD of three independent experiments. * represents *p* < 0.05.

The knockdown of DNER in the gastric cancer cells was accompanied with the upregulation of p21^cip/waf^ (Figure 5C), which may inhibit cell cycle progression. Therefore, the effect of p21^cip/waf^ upregulation in DNER-silenced cells was evaluated by the knockdown of p21^cip/waf^ with shRNA in DOX-inducible shDNER cells. The Western blot data in Figure 10A confirmed the knockdown of p21^cip/waf^ expression in shDNER cells using p21^cip/waf^ shRNA. The knockdown of p21^cip/waf^ expression in shDNER cells resulted in a significant increase in cell viability in 7 days by 46.4% compared to cells with the shSC control (*p* = 0.00059). The recovery reached to 73.8% level of the DOX-untreated shDNER cells (*p* = 0.0061) (Figure 10B). Thus, silencing p21^cip/waf^ did recover the reduction in cell viability caused by DNER silencing partially. Flow cytometric analysis further showed that the S phase cells were increased by p21^cip/waf^ silencing in shDNER cells by 1.61-fold (*p* = 0.038) and were recovered up to 85.6% of the level of the DOX-untreated shDNER cells (*p* = 0.44) (Figure 10C). The sub-G_0_ population was also decreased by 78% by the knockdown of p21^cip/waf^ in shDNER cells (*p* = 0.0011) (Figure 10C). Collectively, silencing p21^cip/waf^ did recover cell viability and restore the cell cycle arrest imposed by DNER silencing, but not as strong as the effects of NICD overexpression. Taken together, these results suggest that p21^cip/waf^ mediates the effect of DNER silencing partially.

### 2.10. Silencing p53 Decreased the Apoptosis of DNER-Silenced Cells

**Figure 11:**
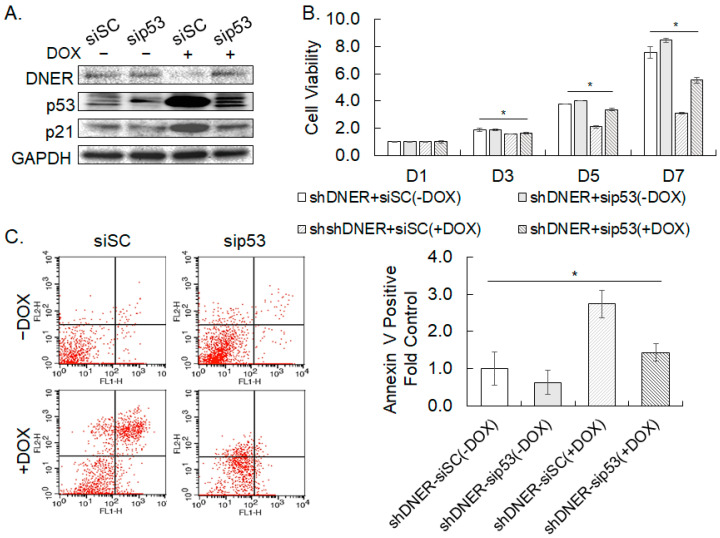
**Silencing p53 decreases the apoptosis of shDNER cells.** (**A**). The level of the indicated proteins, including p53 was examined by Western blotting. The SNU-638 cells transduced with DOX-inducible shDNER were transfected with siSC or sip53 and were then treated with DOX for seven days. GAPDH was used for loading control in the Western blotting. (**B**). The cell viability of the indicated cells was measured using the MTT assay after 1, 3, 5, and 7 days of DOX treatment. Data shown are fold increases over the DOX-untreated shDNER-siSC cells. (**C**). Flow cytometric analysis of Annexin V binding. SNU-638 cells were transduced and treated as shown in Figure 11A. Data shown for Annexin V-positive cells are fold increases over the DOX-untreated siSC cells. All quantitative data shown here are the mean ± SD of three independent experiments. * represents *p* < 0.05.

Silencing DNER expression in the gastric cancer cells upregulated p53 expression (Figure 9A). Hence, in order to investigate its role in cell viability reduction and apoptosis caused by DNER silencing, silencing p53 was attempted by transfection of sip53. Western blot analysis verified the effective silencing of p53 expression (Figure 11A). Silencing p53 expression in shDNER-expressing cells increased cell viability by 1.59-fold in 5 days and by 1.79-fold in 7 days, respectively, but restored it only up to 83.8% of the level in 5 days and 65.2% in 7 days of the DOX-untreated shDNER cells (Figure 11B). The Annexin V-positive population was also found to have decreased to 48% of the shDNER cells by p53 silencing (*p* = 0.006), but not as low as shown in the DOX-untreated shDNER cells (*p* = 0.026, Figure 11B). Knockdown of p53 expression appears to restore cell viability reduction and apoptosis caused by DNER silencing, but not as effective as was previously shown in NICD-overexpressing cells. As a whole, these results highlight the involvement of p53 in the apoptotic process of the gastric cancer cells with DNER knockdown.

### 2.11. Secreted Mouse DNER Interfered with Notch Signaling and Causes Reduction in Cell Viability

**Figure 12:**
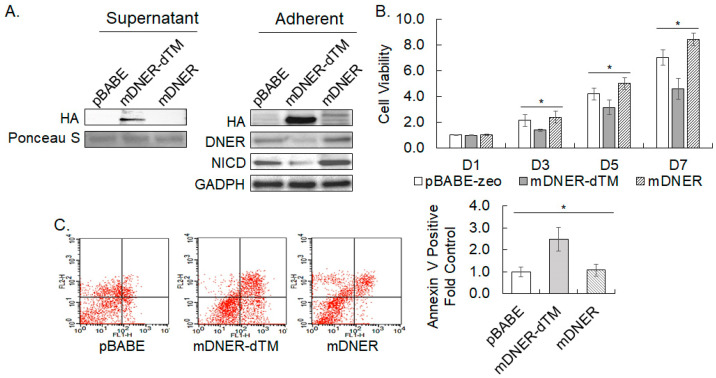
**Membrane-unbound mouse DNER mutant interferes with Notch signaling and causes a reduction in cell viability**. (**A**) Expression of HA-tagged mDNER and mDNER-dTM in the growth medium and within the cells transduced with pBABE-zeo-based retroviruses that were incubated for five days. The secreted protein of mDNER-dTM was collected from serum-free growth medium. Ponceau S was used to monitor the protein loading, while GAPDH was used to monitor the protein loading of the cell lysate. (**B**) The cell viability of cells expressing mDNER or mDNER-dTM was measured using the MTT assay after 1, 3, 5, and 7 days of retroviral transduction. Data shown are fold increases over the cells transduced with the empty vector retrovirus. (**C**) Flow cytometric analysis of Annexin V binding. SNU-638 cells were transduced and treated as in Figure 11A. Data for Annexin V-positive cells are fold increases over the cells transduced with empty vector retrovirus. All quantitative data shown here are the mean ± SD of three independent experiments. * represents *p* < 0.05.

DNER is a transmembrane protein whose location is required for the activation of Notch signaling. Deletion of the transmembrane domain of DNER could interfere with the Notch/DNER interaction and, thereby, Notch signaling, which was examined in this study by the overexpression of a deletion mutant of the transmembrane domain of murine DNER (mDNER-dTM) by retroviral transduction into the gastric cancer cells. The expression of HA-tagged mDNER and HA-tagged mDNER-dTM in the cell lysate was verified by Western blotting (Figure 12A). The expression of mDNER-dTM decreased the protein level of endogenous DNER and NICD to 48.5% (*p* = 0.049) and 38.9% (*p* = 0.008) of the pBABE control, respectively, suggesting that mDNER-dTM effectively blocked Notch signaling. In contrast, the expression of mDNER increased the levels of endogenous DNER (1.37-fold, *p* = 0.084) and NICD (1.45-fold, *p* = 0.090) with a marginal significance. Expression of mDNER-dTM significantly reduced cell viability by 35% (*p* = 0.002), 25% *(p* = 0.005), and 35% (*p* = 0.0001) compared to the vector control on days 3, 5 and 7 of culture, respectively (Figure 12B). On the contrary, cell viability of mDNER-expressing cells was elevated only slightly but meaningfully by 1.19-fold (*p* = 0.013) and 1.20-fold (*p* = 0.001) over the vector control on days 5 and 7, respectively (Figure 12B). Furthermore, the Annexin V-positive population in the mDNER-dTM expressing cells was significantly increased by 2.4-fold (*p* = 0.010) compared to the vector control, while that in mDNER-expressing cells did not reveal a meaningful difference (Figure 12C). Although human DNER was not used in these experiments, murine DNER seemed to be effective to activate Notch signaling, and its transmembrane domain deletion mutant could reduce cell viability and induce apoptosis by interfering with the signaling pathway. These results suggest a possibility that the Notch-interacting domain of DNER could be used to restrict growth and induce the apoptosis of the gastric cancer cells.

### 2.12. TGF-β Signaling Pathway Modulated Spheroid Formation of Gastric Cancer by Targeting DNER

**Figure 13:**
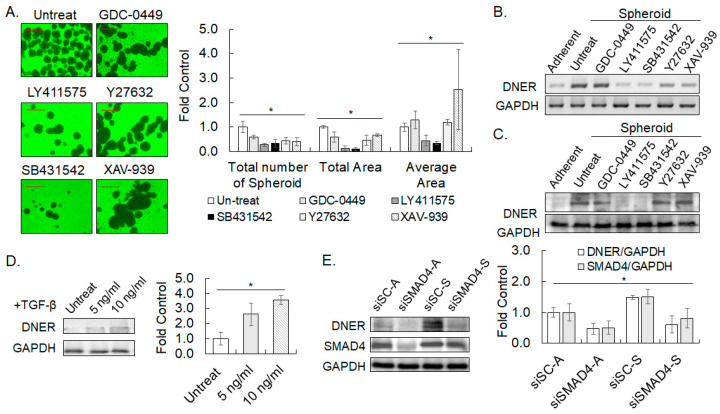
**TGF-β signaling pathway modulates spheroid formation and DNER expression.** (**A**) Phase contrast microscope images (20×) of spheroid formation of SNU-638 after 14 days of spheroid culture in the presence of the indicated inhibitors. A scale bar of 500 µm. Hedgehog inhibitor (GDC-0449, 10 µM); γ-secretase inhibitor for Notch signaling (LY411575, 10 µM); TGF-β inhibitor (SB431542, 10 µM); ROCK inhibitor (Y27632, 10 µM); and tankyrase1/2 inhibitor for Wnt signaling (XAV-939, 10 µM). (**B**,**C**) The DNER transcript level (**B**) and protein level (**C**) in spheroid-cultured SNU-638 cells for 14 days was determined by RT-PCR and Western blotting, respectively. GAPDH was measured to monitor the inputted RNA amount in RT-PCR and to monitor the protein loading in Western blotting. (**D**) DNER protein level in SNU-638 cells treated with an indicated amount of TGF-β for three days was examined by Western blotting. The protein level of DNER normalized against that of GAPDH. The intensity of each protein band was quantitated with ImageJ software. Data shown are fold increases over the untreated control. (**E**). The expression of DNER in SNU-638 cells transfected with siSC or siSMAD4 and grown under adherent (A) and spheroid (S) cultured conditions was examined by Western blotting. The band intensity was quantitated and analyzed as described above. Data shown are fold increases over the siSC-transfected cells grown in adherent conditions. All quantitative data shown here are the mean ± SD of three independent experiments. * represents *p* < 0.05.

Diverse signaling pathways, including TGF-β, Notch, and Wnt were involved in the growth and survival of cells grown under non-adherent conditions and spheroid formation [33,34,35,36]. Thence, specific inhibitors of various signaling pathways were treated, and spheroid formation ability and DNER expression were examined to investigate the signaling pathways involved in the spheroid formation and DNER upregulation by the spheroid culture of the gastric cancer cells. Five inhibitors, namely GDC-0449, LY411575, Y27632, SB431542, and XAV-939 were treated individually to inhibit Hedgehog, Notch, ROCK, TGF-β, and Wnt signaling pathways, respectively. The total number of spheroids, and the total area taken by the spheroid were significantly decreased by the treatment of any of the inhibitors with different potencies (Figure 13A). Among them, LY411575 and SB431542 were the most potent, and exhibited a similar potency. Coincidentally, only LY411575 and SB431542 meaningfully reduced the average size of the spheroids (*p* = 0.026 and *p* = 0.003, respectively). These results imply the critical importance of Notch and TGF-β signaling in spheroid formation of the gastric cancer cells. DNER transcript levels measured by RT-PCR was also reverted to the basal level observed in the adherent cells and declined to 33% *(p* = 0.009) and 31% (*p* = 0.007) of the level of the untreated spheroid-cultured cells by the LY411575 and SB431542 treatments, respectively (Figure 13B). DNER protein levels increased by spheroid culture was significantly curtailed by 72% (*p* = 0.0058) and 76% (*p* = 0.01) by the LY411575 and SB431542 treatments, respectively, and returned to the basal level observed in adherent cells (Figure 13C). Unlike the protein level, a 40% reduction (*p* = 0.039) in DNER transcript level was also observed by the XAV-939 treatment. These results demonstrate that upregulation of DNER expression upon spheroid culture should be dependent on the TGF-β and Notch signaling pathways. In addition, DNER expression was increased by 2.62-fold (*p* = 0.031) and 3.57-fold (*p* = 0.001) by TGF-β treatment at 5 ng/mL and 10 ng/mL, respectively (Figure 13D). Increase in DNER expression by TGF-β treatment was also found in SNU-216, 484, and 668 cells (Supplemental Figure S2). In addition, knockdown of SMAD4 expression with siSMAD4 transfection significantly downregulated DNER expression by up to 40% compared to the scrambled control in both the adherent (*p* = 0.006) and spheroid-cultured (*p* = 0.04) cells, which was comparable to the reduction in SMAD4 level (50% and 53% reduction in each case, respectively) by siSMAD transfection (Figure 13E). Taken together, these findings demonstrate a critical role of TGF-β signaling in the upregulation of DNER expression in both adherent and spheroid-cultured cells. However, the relationship between TGF-β signaling and Notch signaling in spheroid formation and DNER upregulation is not clear at this moment and remains to be determined by future studies.

## 3. Discussion

DNER has been shown to be associated with various pathological parameters and a worse prognosis in gastric cancer patients [28,29]. Moreover, silencing DNER was found to inhibit cell proliferation, migration, and invasion, and induce the apoptosis of gastric cancer cells [28]. The present study investigated the clinicopathological association of DNER expression in gastric cancer tissue, and its potential role and mechanism in the regulation of tumor spheroid formation, cell proliferation, apoptosis, and invasion.

### 3.1. Association of DNER Expression with Clinicopathology of Gastric Cancer

Analysis of the RNASeq data from the TCGA database revealed that DNER expression was significantly correlated with various pathological parameters, including grade, stage, T status, recurrence/progression, new tumor emergence, and survival. The positive correlation of DNER expression with the phenotypes of advanced gastric cancer, recurrent/progressed cancer, and new tumor emergence may contribute to worse overall and disease-specific survival rates of gastric cancer patients. Meaningful associations with tumor size, lymph node metastasis, and poor survival were also reported in a comparative analysis of DNER expression between normal and cancer tissues in 30 patients [28]. In addition, a high expression of DNER was found to be associated with an increased mortality in stomach adenocarcinoma [29]. These results suggest a role of DNER in gastric cancer progression, and the potential to utilize DNER expression as a diagnostic marker. Furthermore, DNER has been reported to be upregulated in various cancer tissues, such as breast and prostate cancer tissues [25,26,37]. In addition, a high expression of DNER was found to be correlated with the tumor grade, recurrence, and overall survival of breast cancer patients [37], and with the tumor stage, M status, and overall survival of hepatocellular carcinoma patients [27]. Moreover, DNER conferred resistance against epirubicin-induced growth inhibition and apoptosis through the Wnt/β-catenin pathway in breast cancer cells [37]. Accordingly, knockdown of DNER expression enhanced the sensitivity to epirubicin in the breast cancer cells [37], and to various drugs in SNU-638 gastric cancer cells (Figure 8A). These findings strongly suggest that DNER expression could be a predictive marker of the chemotherapeutic response. Taken together, DNER clearly plays a critical role in the progression of cancer and patient prognosis, at least in gastric cancer, breast cancer, prostate cancer, and hepatocellular carcinoma.

### 3.2. A Role of DNER in Regulation of Tumor Spheroid Formation, Cell Viability, Cell Proliferation and Apoptosis

Tumor spheroid culture under a special non-adherent condition has been widely used to enrich tumor stem cells and stem cell-like cells, and several gastric cancer cell lines are known to form tumor spheroids [38,39]. In this study, five out of six gastric cancer cell lines formed spheroids or clusters, and the role of DNER in spheroid formation was examined by knocking down its expression. DNER expression was found to be increased in the spheroid-cultured cells at an early time point, with concomitant increases in the NICD and Notch reporter activity (Figure 2 and Figure 3). Furthermore, silencing DNER by siRNA transfection and DOX-inducible shRNA expression resulted in a significant reduction in the spheroid formation of the gastric cancer cells (Figure 4C,F). These observations are consistent with previous research on PC-3 prostate cancer cells, where knockdown of DNER was also shown to decrease spheroid formation [26]. DNER is thought to facilitate the formation of spheroids not only in gastric cancer but also in other types of cancers, including prostate and breast cancer [25,37]. However, it should be noted that inhibition of glioblastoma-derived neurosphere formation by DNER was reported previously [40], thereby suggesting that caution should be placed in generalizing the effect of DNER expression on tumor spheroid formation. In addition, DNER expression was also increased in SNU-216, which did not form obvious cell clusters or spheroids (Figure 2). This result raises questions regarding the mechanisms of DNER-facilitated spheroid formation. Whether DNER directly contributes to spheroid formation by aggregating cells into a cluster, or to promoting the proliferation and survival of cells grown in the non-adherent condition, remains to be determined.

DNER was proposed to promote cell proliferation and inhibit the apoptosis of breast, prostate, and gastric cancer cells, which could be a mechanism of DNER-facilitated spheroid formation [25,26,28,37]. Silencing DNER expression reduced cell viability and inhibited cell cycle progression of the gastric cancer cells studied here. DNER-silenced cells accumulated in the G_1_ phase, and were inhibited from progressing into the S phase, indicating a G_1_ arrest in the absence of DNER (Figure 5A,B). The sub-G_0_ cell population was also increased by DNER silencing. Reduction in S phase cells with a simultaneous increase in G_1_ phase cells by silencing DNER was also observed in PC-3 prostate cancer cells [26]. On the contrary, downregulation of DNER elicited an increase in S phase cells with a corresponding decline in G_1_ phase cells in MDA-MB-231 cells, but a decrease in S phase cells with an increase in G_2_/M phase cells in MDF-7 cells was also observed [25]. Therefore, DNER silencing did inhibit cell cycle progression in these cells, but its effect on the specific cell cycle phase appeared to be dependent on the cell type.

The expression of p21^cip/waf^, p27, and p53 was found to be upregulated in DNER-silenced gastric cancer cells (Figure 5C). It is known that p53 can transactivate p21^cip/waf^ and p27, and that p21^cip/waf^ and p27 can inhibit cyclin-dependent kinase, thereby inhibiting cell cycle progression [30,31,41]. Thus, p21^cip/waf^ and p27 induced by p53-mediated transactivation might be responsible for the observed cell cycle arrest. Indeed, knockdown of p21^cip/waf^ expression resulted in a significant increase in cell viability and S phase cells at the expense of G_1_ phase cells (Figure 10). Furthermore, the expression of p21^cip/waf^ was found to have decreased due to silencing p53 expression, as shown in Figure 11. However, silencing p21^cip/waf^ only partially restored the reduction of cell viability and S phase cells observed in DNER-silenced cells, to a level of 73.8% and 85.6% of that observed in DNER-expressing cells, respectively. Therefore, the upregulation of p21^cip/waf^ expression should be considered as one of the factors contributing to the observed cell cycle arrest in DNER-silenced cells.

Apoptosis is known to be another factor that can reduce cell viability of DNER-silenced breast, gastric, and prostate cancer cells. In the gastric cancer cells, knockdown of DNER expression induced apoptosis, but not necrosis (Figure 6A,B). It has been reported that Notch-1 can inhibit p53-mediated apoptosis through the PI3K-AKT-mTOR pathway [32,42]. Inhibition of PI3K-AKT signaling by DNER silencing has been proposed in the regulation of cell viability and apoptosis of breast cancer cells and hepatocellular carcinoma [25,27]. Knockdown of p53 expression partially restored cell viability and decreased apoptotic cell death in DNER-silenced gastric cancer cells, but not to the level observed in DNER-expressing cells (Figure 11). If cell survival and proliferation promoted by DNER were solely mediated by PI3K-Akt, additional signals from the PI3K-Akt pathway activating cell survival and proliferation should also be considered [43].

Distinct caspases were activated depending on the culture conditions in DNER-silenced gastric cancer cells. While cleaved caspases 3 and 8 were detected in DNER-silenced cells grown under both adherent and spheroid culture conditions, cleaved caspase-9 was only increased in adherent cells (Figure 6C). In addition, inhibition of caspase-9, but not caspase-8, reduced the level of cleaved caspase-3 in adherent cells (Figure 7B). Unexpectedly, however, inhibitors of caspases 8 and 9 were similarly effective in preventing the apoptosis of DNER-silenced cells grown under adherent conditions (Figure 7A). Based on these results, the activation of caspase 8 followed by caspase 3 appears to be the major pathway of apoptosis in DNER-silenced cells grown in spheroid culture. Caspase-9 seems to play a dominant role in the apoptosis of adherent DNER-silenced cells. However, it is not yet clear how caspase-8 induces apoptosis in DNER-silenced adherent cells without caspase-3 activation.

Silencing DNER enhanced the sensitivity of gastric cancer cells to various chemotherapeutic drugs (Figure 8A). This finding was consistent with a previous study demonstrating that DNER expression rendered resistance against epirubicin treatment to MCF-7 and MDA-MB-468 breast cancer cells [37]. DNER silencing strongly potentiated the drug effect of all five reagents. It is possible that the inactivation or depletion of DNER could cooperate with the conventional and targeted chemotherapeutics. In that sense, it is interesting that the expression of a membrane-unbound murine DNER mutant effectively reduced cell viability and induced the apoptosis of the gastric cancer cells (Figure 12B,C). These results suggest the feasibility that membrane-unbound species of DNER or Notch-interacting EGF domains of DNER could be developed into cancer drug candidates.

Silencing DNER inhibited the invasion of gastric cancer cells (Figure 8B). In addition, knockdown of DNER expression was shown to inhibit the invasion of breast, prostate, and gastric cancer cells. These results clearly support the involvement of DNER in tumor invasion probably by the induction of epithelial–mesenchymal transition (EMT) as shown in breast cancer cells [37]. Invasion of tumor cells is associated with metastatic capacity that is developed in the advanced stages of cancer. Indeed, DNER expression was associated with the M status of gastric cancer and hepatocellular carcinoma. However, DNER expression was not found to be significantly associated with the M status in this study, potentially due to biases in patient numbers (337 M0 vs. 19 M1, respectively). Furthermore, DNER expression in breast cancer tissue was not meaningfully associated with lymph node metastasis in two independent studies either [25,37]. The association of DNER expression with tumor metastatic status might therefore be dependent on the type of cancer.

### 3.3. Regulation of DNER Expression by the TGF-β Signaling

TGF-β signaling has been known to regulate tumor spheroid formation in breast and pancreatic cancer cells [44,45,46]. Inhibition of TGF-β signaling significantly interfered not only with the spheroid formation of the gastric cancer cells, but also with the upregulation of DNER in the spheroid-cultured cells (Figure 13A–C). The upregulation of DNER in the spheroid-cultured cells was also inhibited by the knockdown of SMAD4 expression, a mediator of TGF-β signaling (Figure 13E). Therefore, DNER could be one of the mediators of the TGF-β signal that promotes tumor spheroid formation. Notch signaling also regulates the spheroid formation and expression of DNER in the spheroid-cultured cells (Figure 13A–C). Since DNER is involved in spheroid formation (Figure 4), Notch signaling activated by DNER, which was upregulated by TGF-β, should therefore regulate the spheroid formation. One question raised here is how Notch signaling regulates DNER expression. Overexpression of NICD increased DNER expression in the gastric cancer cells (Figure 9A), suggesting a positive feedback regulation of Notch signaling. Such positive feedback among the DNER-Notch-NICD loop would explain the involvement of Notch signaling in DNER upregulation in the spheroid-cultured cells. On the other hand, shDNER-mediated DNER silencing was recovered by the silencing of p53 expression (Figure 11A), which suggests a cross-talk between the p53 signal and Notch signaling [47,48,49], indicating a complex interplay between multiple signaling pathways.

### 3.4. DNER in the Notch Signaling

Activation of Notch signaling regulates cancer cell proliferation, survival, and differentiation through the production of NICD [50,51,52]. DNER is a DSL-deficient non-canonical Notch ligand that is known to bind to Notch1 and act as a ligand for it [20]. However, a recent study reported contradictory results, suggesting that DNER does not bind to Notch1 and activate Notch signaling [21]. In this study, the elevation of DNER expression was accompanied with an increase in NICD protein level in the spheroid culture of gastric cancer cells (Figure 3B). Additionally, knockdown of DNER expression resulted in the downregulation of NICD and Notch reporter activity (Figure 4G, Figure 5C and Figure 6C). These results support the notion that DNER functions as a specific ligand of Notch1. Moreover, the overexpression of NICD reversed the decrease in cell viability, inhibition of cell cycle progression, and induction of apoptosis caused by DNER silencing, further supporting the function of DNER as a specific Notch ligand. The reduction in DNER expression followed by the suppression of NICD level and Notch reporter activity, as well as the reversion of the effects of DNER silencing by the ectopic expression of NICD, provides compelling evidence that NICD, produced by the activation of the Notch signal, mediates the biological effects of DNER in the gastric cancer cells.

## 4. Materials and Methods

### 4.1. Gastric Cancer Cell Lines and Cell Culture

The gastric cancer cell lines, SNU-216, SNU-484, SNU-601, SNU-638, SNU-668, and SNU-719 were all purchased from the Korea Cell Line Bank (Seoul, Republic of Korea). The RPMI-1640 (Gibco, Grand Islands, NY, USA) medium supplemented with 10% fetal bovine serum (FBS) (Welgene, Daegu, Republic of Korea), 5% L-glutamine (Gibco), and 1% penicillin/streptomycin (Gibco) was used for the cell culture. The gastric cancer cells were cultured at 37 °C in a humidified-5% CO_2_ incubator and were sub-cultured twice per week.

### 4.2. Spheroid Formation Assay

Gastric cancer cells were cultured in a non-adherent condition with an ultralow attachment 6-well plate (Corning, Corning, NY, USA). The cells were seeded at 3 × 10^3^ cells per well. The medium for the spheroid culture was RPMI-1640 containing 1% N-2 supplement (Gibco), 2% B-27 supplement (Gibco), 20 ng/mL human FGF-2 (ProSpec, East Brunswick, NJ, USA), 100 ng/mL EGF (ProSpec), and 1% penicillin/streptomycin (Gibco). Tumor spheroid formation was monitored with a phase contrast microscope at 20× magnification (Olympus, Tokyo, Japan). ImageJ software (Ij153-win-java8, https://imagej.nih.gov, accessed on 23 June 2021) was used for the analysis of the spheroid number and the area taken by the spheroids.

### 4.3. Luciferase Assay

Cells were seeded at 10^4^ cells per well on 12-well plates on the prior day of transfection. Notch promoter reporter (0.9 µg 6XNRE-Luc, [53])and pRL-TK (0.1 µg Promega, Madison, WI, USA) were co-transfected to each well (100 µL each well) with Lipofectamine 2000^®^ transfection reagent following the manufacturer’s instructions (Life Technologies, Carlsbad, CA, USA). Transfected cells were cultured either in adherent condition or in spheroid culture condition for three days and were then harvested with passive lysis buffer (Promega). Doxycycline-inducible shDNER cells were transfected with the Notch reporter as above. After 24 h, doxycycline (DOX, 10 ng/mL) was treated and incubated for an additional three days and harvested with the passive lysis buffer. The harvested cells were incubated for 15 min with shaking at RT. The lysate was then centrifuged at 13,000 rpm at 4 °C for 10 min, and the supernatant was collected. The supernatant (10 µL) was used for luciferase activity measurement by the Dual Luciferase^®^ Reporter Assay System (Promega) with a Lumat LB 9507 Luminometer (Berthold Technologies, Oak Ridge, TN, USA).

### 4.4. Invasion Assay

A 24-well transwell plate with an 8 μm pore size (SPL Life Sciences, Pocheon, Republic of Korea) was used to measure cell migration and invasion capacity. The upper chamber of the transwell plate was coated with 100 µL of Matrigel^®^ (100 μg/µL in serum-free RPMI-1640 medium, BD Biosciences, Sparks, MD, USA). A total of 2.5 × 10^5^ cells were resuspended in serum-free RPMI-1640 medium and then added to the upper chamber. The lower chamber was filled with RPMI-1640 medium supplemented with 1% FBS. Cells were incubated for 12 h and then fixed with 3.7% formaldehyde (Duksan Reagent, Seoul, Republic of Korea) in phosphate-buffered saline (PBS). The fixed cells were stained with 0.5% crystal violet solution (Sigma-Aldrich, St. Louis, MO, USA). After removing the staining solution, cells were washed twice with PBS and dried in the chamber of the transwell plate overnight. Cotton swabs soaked with water were used to clean the membrane on the upper chamber. The cell number was counted under the microscope (20×) (Olympus, Tokyo, Japan). The number of cells was counted in a total of 4 fields of the plate.

### 4.5. Cell Viability Assay

Cell viability was measured using the 3-(4,5-dimethylthiazol-2-yl)-2,5-diphenylterazolium bromide (MTT, Sigma-Aldrich) assay [54]. After removing the media, 100 µL of MTT mixture (0.5 mg/mL MTT in culture medium) was added to each well of the 96-well plate. After incubation for 3 h, 100 µL of MTT solubilizer (10% SDS in 0.01 N HCl) was added to each well and incubated at 37 °C overnight to dissolve the formazan crystal. The absorbance at 570 nm with a baseline absorbance at 650 nm was then measured using a Multiskan GO spectrophotometer (Thermo Scientific, Rockland, IL, USA).

### 4.6. Western Blot Analysis

Cells lysates were prepared using a radioimmunoprecipitation (RIPA) buffer containing 50 mM Tris-HCl (pH 7.4), 0.1% SDS, 1% Triton-X-100, 0.1% Nonidet P-40, and 0.5% sodium deoxycholate. The buffer was freshly supplemented with 1 mM DTT and protease inhibitor cocktail (Pierce Biotechnology, Rockford, IL, USA). The BCA protein assay (Pierce Biotechnology) was used to measure the protein concentration, and 30 µg of protein was loaded per well onto an SDS-polyacrylamide gel (8 or 10%). The proteins were then transferred to a PVDF membrane (Roche, Penzberg, Germany), and the membrane was blocked with 5% nonfat-dried milk in TBST (10 mM Tris HCl [pH 7.6], 150 mM NaCl, and 0.1% Tween 20) for 1 h at RT. After overnight incubation with primary antibodies (1:2000 dilution in TBST-1% nonfat-dried milk), the membrane was washed and incubated for 1 h with HRP-conjugated anti-rabbit or anti-mouse secondary antibody at RT (1:10,000 dilution in TBST-1% nonfat-dried milk, Pierce Biotechnology). The primary antibodies used were αDNER (Santa Cruz Biotech, Dallas, TX, USA), αNOTCH (Santa Cruz Biotech), αCaspase3, αCaspase8, αCaspase9, αTP53, αp21, and αGAPDH (all from Cell Signaling Tech, Danvers, MA, USA).

### 4.7. Flow Cytometry

Cells were harvested by trypsinization and washed twice with PBS. For cell cycle analysis, the cells were fixed and permeabilized with 70% cold ethanol and stained with PI (0.05 mg/mL, Sigma-Aldrich) for 30 min. Apoptosis was determined with the cells stained by FITC-Annexin V for 30 min (BD Bioscience). PI (final concentration of 0.05 mg/mL) was added to the FITC-Annexin V-stained cells and was analyzed with flow cytometry immediately. BrdU (BD Bioscience) was used to measure newly synthesized DNA. Cells were pulsed with 20 µM BrdU for 2 h following which the cells were harvested and washed twice with PBS. After fixation and permeabilization with 70% cold ethanol for 15 min followed by DNA fragmentation with 2 N HCl at RT for 30 min, the cells were resuspended with 0.1 M Na_2_B_4_O_7_ for 2 min. Then, the cells were divided into two aliquots and stained with FITC-α-BrdU and the FITC-mouse IgG_1_ isotype control in the dark for 45 min, respectively. After that, 7-AAD (Sigma-Aldrich, final concentration of 1.5 µg/mL) was added for staining. The fluorescent signal was measured with flow cytometry with FACSCalibur^TM^ (BD Bioscience) and analyzed with Quest Pro^TM^ software (5.2.1 version, BD Bioscience) and with ModFit LT^TM^ (version 3.1, Verity Software House).

### 4.8. Knockdown of Gene Expression by Lentiviral Transduction or RNA Interference

The expression of p21^cip/waf^ was silenced by the transduction of pLKO-puro-based shRNA lentivirus [32], while that of DNER was knocked down by the transduction of Tet-pLKO-puro-based shRNA lentivirus followed by DOX treatment (10 ng/mL for final concentration). Scrambled and DNER-specific shRNA-coding sequences (Supplementary Table S1) were inserted at the Age1/EcoR1 double-digested site of the Tet-pLKO-puro (Addgene, Cambridge, MA, USA). Production and transduction of the lentiviruses were performed as previously described by To et al. (2023) [38].The gastric cancer cells transduced with the lentiviruses were selected with puromycin (2 µg/mL) for seven days and were used for further experiments. The expression of p53 and DNER was silenced by the transfection of gene-specific siRNAs (Supplementary Table S1) [37,55]. Scrambled siRNA or a gene-specific siRNA was transiently transfected into the gastric cancer cells with the Lipofectamine 2000^®^ transfection reagent (Life Technologies) as according to the manufacturer’s instructions.

### 4.9. Overexpression of mDNER, mDNER-dTM, and NICD by Retroviral Transduction

pBABE-zeo (Addgene) was used as a parental vector for overexpressing murine DNER (mDNER), mDNER-dTM (deletion of the transmembrane domain), or NICD. The HA-tagged-mDNER fragment (aa 25-737) was obtained by the *Eco*R1/Xho1 digestion of pDisplay-DNER (Addgene) and was then cloned into the *Eco*R1/*Sal*1 site of pBABE-zeo. The HA-tagged-mDNER fragment (aa 25-572) with deletion of the transmembrane domain was obtained by the *Eco*R1/*Sma*1 digestion of pDisplay-DNER and was cloned into the *Eco*R1/blunted *Sal*1 site of pBABE-zeo. The NICD insert fragment (aa 1755-2556) was obtained by single cutting with *Bam*HI from pCMV4-Flag-NICD and was cloned into the *Bam*H1 site of pBABE-zeo. The production and transduction of the retroviruses were performed as previously described in To et al. [38].The retrovirus-transduced cells were selected by zeocin treatment (100 µg/mL) for seven days.

### 4.10. Statistical Analysis

RNASeq data of gastric cancer tissue was retrieved from the TCGA database (Stomach adenocarcinoma, PanCancer Atlas) through the cBioPortal (http://www.cbioportal.org/, accessed on 1 April 2023). The data were analyzed using the Wilcoxon rank sum test, Kaplan–Meier survival analysis, and log-rank test by the R project (version 4.2.3). Experimental data were analyzed by the two-tailed Student’s *t*-test and one-way ANOVA implemented in Microsoft Excel. A *p*-value less than 0.05 was considered statistically significant.

## 5. Conclusions

DNER expression was associated with the pathological parameters of the advanced stages of gastric cancer and poor patient survival, positioning it as a potential prognostic marker and therapeutic target for clinical intervention. This study provides evidence for the critical role of DNER in regulating spheroid formation, cell proliferation, and survival of gastric cancer cells. DNER silencing evoked a reduction in cell viability resulting from cell cycle arrest, along with the induction of apoptosis in gastric cancer cells. The cell cycle arrest and apoptotic cell death caused by DNER silencing were at least partially attributed to the upregulation of p53 and p21^cip/waf^. The study results also demonstrate that NICD, a key component of Notch signaling, should be a major mediator of DNER’s biological function. DNER, which was upregulated through the activation of TGF-β signaling, is supposed to modulate the spheroid-forming capacity by regulating cell proliferation and the survival of gastric cancer cells. Future research and validation, including in vivo animal models, is necessary to fully elucidate the mechanisms and clinical implications of DNER in gastric cancer.

## Data Availability

The data will be available on request.

## Supplementary Materials

The following supporting information can be downloaded at: https://www.mdpi.com/article/10.3390/ijms241210077/s1.
