# Supplementary material for: Delta/Notch-like Epidermal Growth Factor-Related Receptor (DNER), a Potential Prognostic Marker of Gastric Cancer Regulates Cell Survival and Cell Cycle Progression
Source: Int J Mol Sci. 2023 Jun 13;24(12):10077. doi: 10.3390/ijms241210077 (PMC10298686; doi:10.3390/ijms241210077)
Supplement: Supplementary file 1 [file ijms-24-10077-s001.zip › Supplemental Figures.pdf]

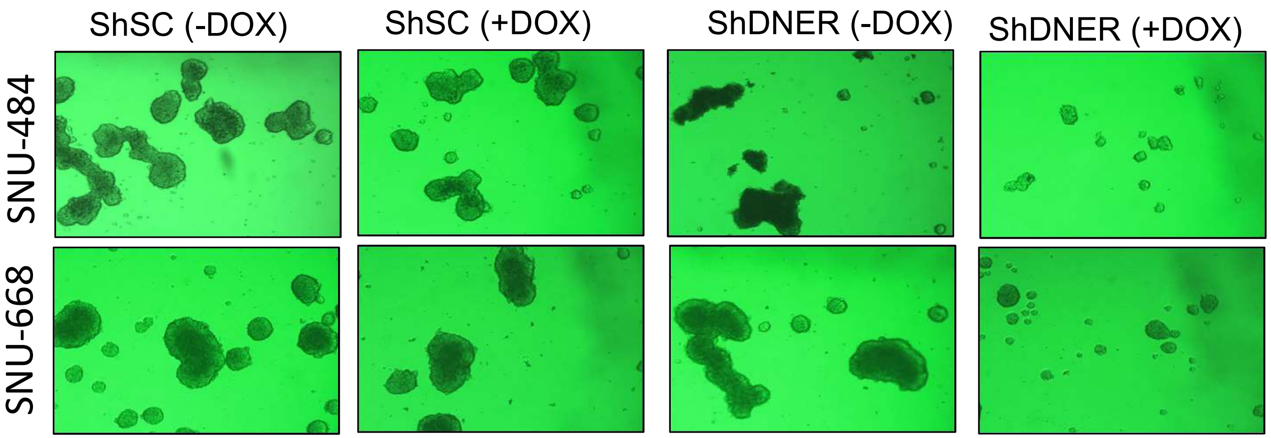

Supplemental Figure S1. Phase contrast microscope images (20x) of spheroid-cultured SNU-484 and SNU-668 cells expressing shSC or shDNER for 14 days. SNU-484 and SNU-668 cells were transduced with lentiviruses expressing shSC or shDNER and were then selected by puromycin (2 µg/ml) treatment for seven days. The expression of shSC or shDNER was induced by doxycycline treatment (10 ng/ml).

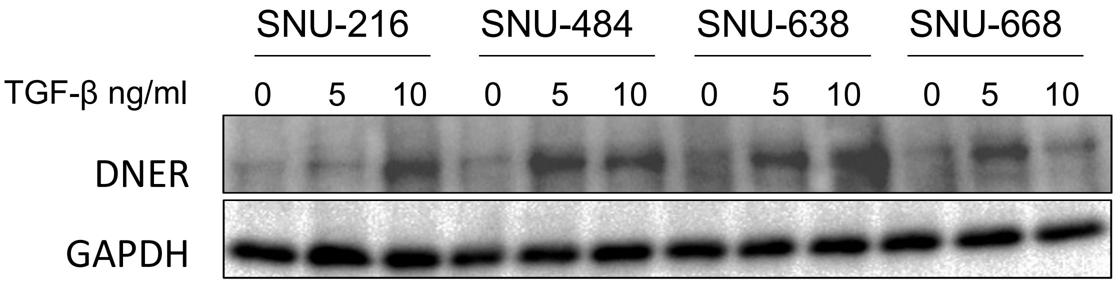

Supplemental Figure S2. DNER protein level in SNU-216, SNU-484, SNU-638 and SNU-668 cells treated with an indicated amount of TGF-β for three days was examined by western blotting. GAPDH was used as a loading control.
