# Supplementary material for: Delta/Notch-like Epidermal Growth Factor-Related Receptor (DNER), a Potential Prognostic Marker of Gastric Cancer Regulates Cell Survival and Cell Cycle Progression
Source: Int J Mol Sci. 2023 Jun 13;24(12):10077. doi: 10.3390/ijms241210077 (PMC10298686; doi:10.3390/ijms241210077)
Supplement: Supplementary file 1 [file ijms-24-10077-s001.zip › Supplementary Table.pdf]

Supplemental Table S1

**Table of Primer**

| **Primer** | **Sequences** |
|---|---|
| DNER F | GTGCCTGGCAGAATACAAAGG |
| DNER R | ATGATGTAGAGGGAGTGCCG |
| NOTCH1 F | CTGAAGAACGCTTCAGACGG |
| NOTCH1 R | ATCAGAGCGTGAGTAGCGG |
| GAPDH F | TGATGACATCAAGAAGGTGGTGAAG |
| GAPDH R | TCCTTGGAGGCCATGTGGGCCAT |
| **siRNA** | |
| siDNER | Sense-GCUUUGCCAGUCCAAGAUU |
| | Anti sense-GCUUUGCCAGUCCAAGAUU |
| siP53 | Sense- AAGGAAAUUUGCGUGUGGAGUUU |
| | Anti sense-ACUCCACACGCAAAUUUCCUUUU |
| siSMAD4 | Sense-AAGAUCUACCCAAGUGCAUAU |
| | Antisense-AUAUGCACUUGGGUAGAUCUU |
| **shRNA** | |
| shDNER | Top: 5′-CCGGGCTTTGCCAGTCCAAGATTGACTCGAGTCAATCTTGGACTGGCAAAGCTTTTTG |
| | Bot: 5′-AATTCAAAAAGCTTTGCCAGTCCAAGATTGACTCGAG TCAATCTTGGACTGGCAAAGC |
